# Transfer learning from inorganic materials to ivory detection

**DOI:** 10.1038/s41598-025-98915-y

**Published:** 2025-05-03

**Authors:** Agil Aghasanli, Plamen Angelov, Dmitry Kangin, Jemma Kerns, Rebecca Shepherd

**Affiliations:** 1 School of Computing and Communications, Lancaster University, Bailrigg, Lancaster, Lancashire LA1 4YW UK; 2 Lancaster Medical School, Lancaster University, Bailrigg, Lancaster, Lancashire LA1 4AT UK; 3 Bristol School of Anatomy, University of Bristol, 32 Southwell Street, Bristol, Avon BS2 8EJ UK

**Keywords:** Machine learning, Data acquisition

## Abstract

This paper describes the automatic identification of ivory using Raman spectroscopy data and deep neural network (DNN) models pre-trained on open-source data from inorganic minerals. The proposed approach uses transfer learning (TL) from foundation models trained on a larger inorganic (minerals) spectroscopy dataset (MLROD). The results demonstrate, for the first time, the ability to transfer machine learning (ML) models from a Raman spectroscopy dataset of geological substances to classify biological ivory samples. Current identification methods, such as DNA analysis and radiocarbon dating, are costly and destructive. Recently, it was demonstrated that the use of Raman spectroscopy, a laser-based, non-destructive technique, in combination with well-known statistical techniques, has the potential to differentiate between mammoth and elephant ivory. However, this previous study had a small sample size due to difficulties in obtaining large amounts of labeled ivory data. To date, there has been no reported work on ivory classification using DNNs, and only limited studies using Raman spectra. The work proposed in this paper suggests that ML can provide high levels of accuracy in the classification of Raman spectroscopy data from ivory samples of different elephant species (up to 99.7%). This has the potential to create a quick and inexpensive method of identifying legal and illegal types of ivory to aid in enforcement of ivory trade bans. This study also demonstrated that DNN models initially pre-trained on inorganic minerals (from the MLROD dataset) that were not finetuned on ivory data had a high accuracy rate of 92%, alleviating the need for large amounts of training data from ivory specimens. Finally, the approach proposed in this paper, provides insight into the decision making and interpretation of the results using prototype-based models. This novel work demonstrates that: (1) ML methods can provide highly accurate classification of ivory from different species of elephant using data obtained using Raman spectroscopy and providing insight into the decision making (2) TL enables re-purposing the models trained on larger mineral datasets of inorganic materials (such as MLROD) to discriminating between the classes of ivory, containing inorganic and organic biological components, for the first time transgressing between non-biological and biological samples (3) the proposed method allows both training from labelled samples of ivory and the identification of unknown ivory samples through prototype-based methods.

## Introduction

This paper describes the automatic identification of ivory samples from extant and extinct species of elephant using Raman spectroscopic data and highly accurate (up to 99.7%) deep neural network (DNN) models pre-trained on Raman data from geological minerals (composed of inorganic materials) and then finetuned to biological ivory samples (a combination of organic and inorganic substances^1^). The importance of such identification stems from the fact that the global trade in ivory has been a crucial contributing factor to the decline in elephant numbers worldwide.

There are three living species of elephant, *Loxodonta* *africana* (the African Savannah elephant), *Loxodonta* *cyclotis* (the African forest elephant) and *Elephas* *maximus* (the Asian elephant^2^). While there are global trade bans on ivory from these living species^3^, most countries do not have the same restrictions on the trade of extinct mammoth ivory (primarily *Mammuthus primigenius*)^4^. Global warming is accelerating the thawing of the Siberian permafrost, revealing perfectly preserved mammoth carcasses and increasing the lucrative trade of ‘mammoth hunting’^5^. The rise in sale of mammoth ivory poses problems for law enforcement worldwide, as once tusks become worked into objects, it becomes difficult to distinguish between illegal and legal sources of ivory^6^.

The current techniques used for identification of species of ivory objects include DNA analysis and radiocarbon dating^7^. These specialist techniques are expensive and require the destruction of a portion of the sample in order to generate results^8,9^. While some DNA analysis protocols may be completed in a few days^8^, the overall process, including sample acquisition, pretreatment, analysis, and reporting, can often take weeks or even months depending on logistical and practical constraints. In contrast, Raman spectroscopy, a laser-based, non-destructive technique, offers an alternative that does not require pretreatment and preserves the sample for further analysis or reuse, making it a highly suitable option for rapid identification of ivory. Recent research^6^ has demonstrated that this method, when coupled with Principal Component Analysis (PCA), has potential to be used effectively in ivory identification.

Raman spectroscopy is a method of measuring and quantifying changes in energy of a light (using a laser) as it is inelastically scattered from a material^10^. As the light (photons) interact with molecular bonds, providing energy for them to vibrate, energy is lost or gained, which results in a shift in wavenumber. Raman spectroscopy measures energy shifts in scattered light to produce material-specific spectra, or ’fingerprints’. Biochemical components can be identified, and subsequent multivariate analysis can be used to determine where there are spectral, and therefore biochemical, differences between samples^11^.

Inorganic Raman spectroscopy data, such as the MLROD dataset^12^, contain single mineral specimens and binary powder mineral mixtures. In contrast, ivory is comprised of both organic and inorganic components^6^. The availability of large scale Raman datasets of biological materials are limited. However, the use of an inorganic dataset and TL shows great potential and is proposed for the first time in this paper. When examining Raman spectral peaks from pure inorganic materials, they typically have a distinct peak with a narrow bandwidth^13^. Biological materials are more complex, resulting in a range of chemical environments and wider peaks^14^. Ivory is primarily composed of dentine, a combination of organic collagen and inorganic mineral, specifically, calcium phosphate based dentinal apatite crystals^15^. The Raman spectra of ivory demonstrates identifiable peaks at phosphate (960 $\hbox {cm}^{-1}$), amide I (1665 $\hbox {cm}^{-1}$), amide III (1240 and 1270 $\hbox {cm}^{-1}$), carbonate (1070 $\hbox {cm}^{-1}$) and CH_2 (1450 $\hbox {cm}^{-1}$) peaks^6^. Previous work suggested that the largest differences between the ivory species was attributable to changes within the phosphate peak (960 $\hbox {cm}^{-1}$).

ML techniques, such as deep learning (DL)^16,17^ demonstrated enormous potential in various areas of application, most notably in image processing^18^ and natural language processing^19^, but, more recently also to spectroscopy including both, organic^20^ and inorganic materials^21^. There are a wide range of uses for ML learning techniques in the classification of Raman spectra. Examples include the use of t-distributed stochastic neighbor embedding (t-SNE) with back propagation neural network (BPNN) for use of Raman analysis and classification of peanut oils^22^. With the advent of handheld Raman spectrometers, there has also been the use of ML for compound classification in portable instruments; kNN, naïve bayes (NB), RF, SVM, NN, and CNN have been used on on pure drug spectra, binary, ternary and quaternary mixtures for high accuracy classification^23^. CNN has also been used to analyze the spectra of acrylic paints^24^. To date, however, there is no study specifically on ivory, tusks or bones, although these are tissues that have been characterised by Raman spectroscopy^6^.

Many of these previously used ML techniques have limited explainability, meaning that the user cannot be sure how the data has been interpreted. The emerging field of eXplainable (and interpretable) Artificial Intelligence (xAI) attempts to address this issue^25,26^. Many methods rely on *post hoc* explanations^25,27^, which do not necessarily reflect the actual decision making process. Alternatively, *ante hoc* methods could be used^28,29^, which, by contrast, design the system with explanations in mind. DL techniques may contain millions or even billions of parameters^30^. In order to optimise these parameters, training on large amounts of data is usually required^31^. However, this is not always feasible in fields with limited data sources.

**Fig. 1:**
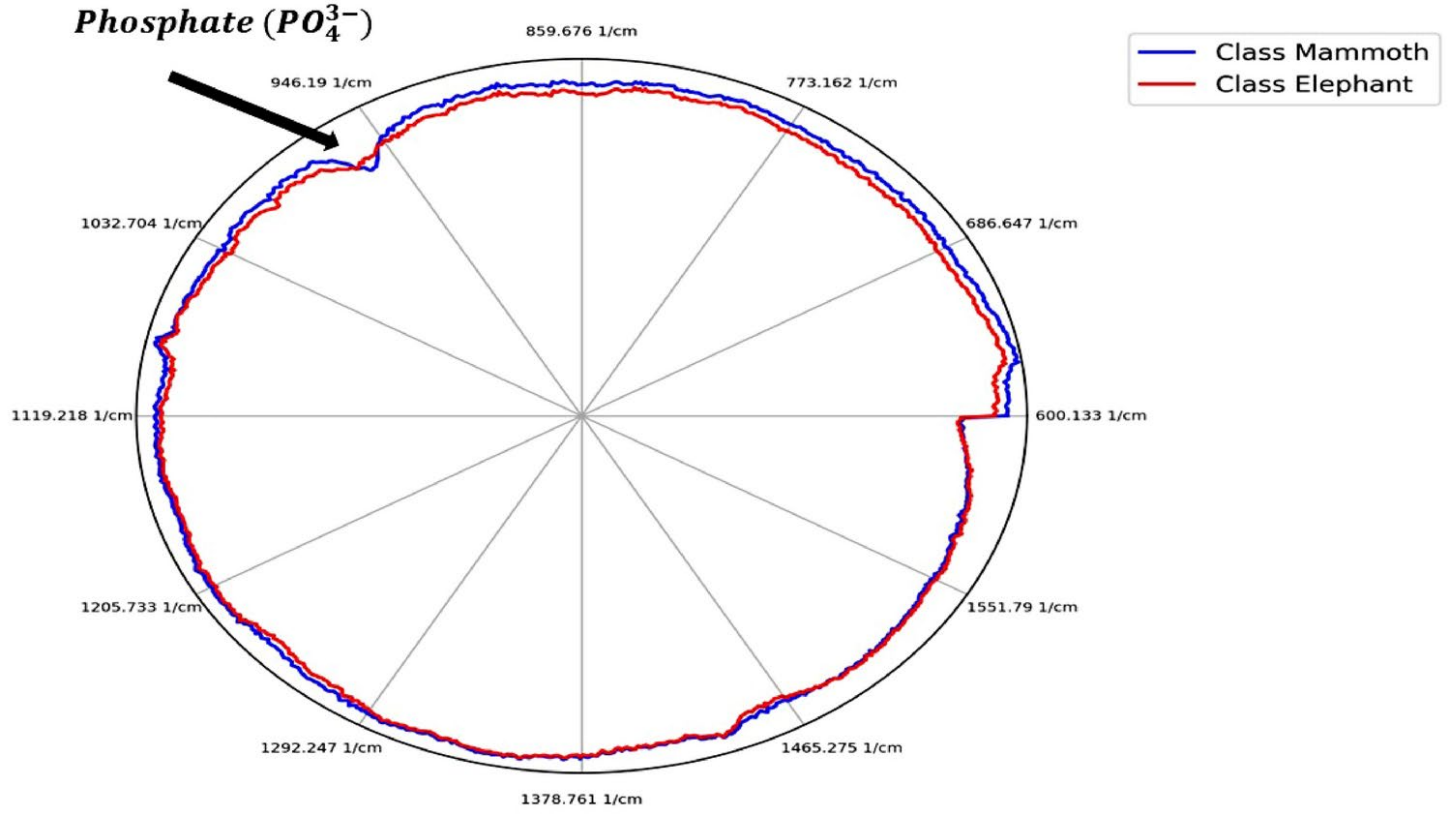
The radar plot shows the distance between the query sample and the nearest prototypes for Mammoth (blue) and Elephant (red) across different wavenumbers. Although the query sample (the origin of the coordinate system) is generally closer to the Elephant prototype, this reverses around the 960 $\hbox {cm}^{-1}$ band, and results in a correct classification as Mammoth. Thus, the spectrum values at 960 $\hbox {cm}^{-1}$ play a key role in the decision.

TL techniques^32^ allow for the re-use of existing models trained on other datasets, with a goal of reducing training time by leveraging the models trained on larger amounts of data. These techniques are of increasing importance with the advent of foundation models, such as CLIP^33^ or DinoV2^30^. These are pre-trained on large amounts of data for enhanced generalisation across a range of tasks. This work introduces this idea into the domain of Raman spectroscopy, demonstrating, for the first time, that models trained on hundreds of thousands of inorganic data samples can be used to identify completely different type of data—in this case, obtained from extant and extinct ivory samples. This work: (1) proposes a ML method which can provide accuracy of ivory discrimination up to $99.7\%$ using Raman spectroscopy data, while also providing insight into the decision making; (2) introduces for the first time the use of TL from DL models trained on larger and open source geological datasets for discriminating between different species of ivory; (3) offers an approach that does not require labeling organic ivory data through the reliance on the TL and DL model pre-trained on large inorganic data which gives accuracy of up to $92\%$. The latter alternative while slightly less accurate than the former one ($92\%$ versus $99.7\%$) is advantageous due to the difficulties in obtaining large amounts of Raman spectra from previously identified elephant species. This is important when identifying the species of previously unclassified ivory. This work also demonstrates that our model emphasizes the 960 $\hbox {cm}^{-1}$ phosphate band when making classification decisions. This is evidenced by the radar plot shown in Fig. 1, where the query sample is correctly classified based on its proximity to the according prototype in this region.

## Results

### Data

#### Ivory classification dataset

**Fig. 2:**
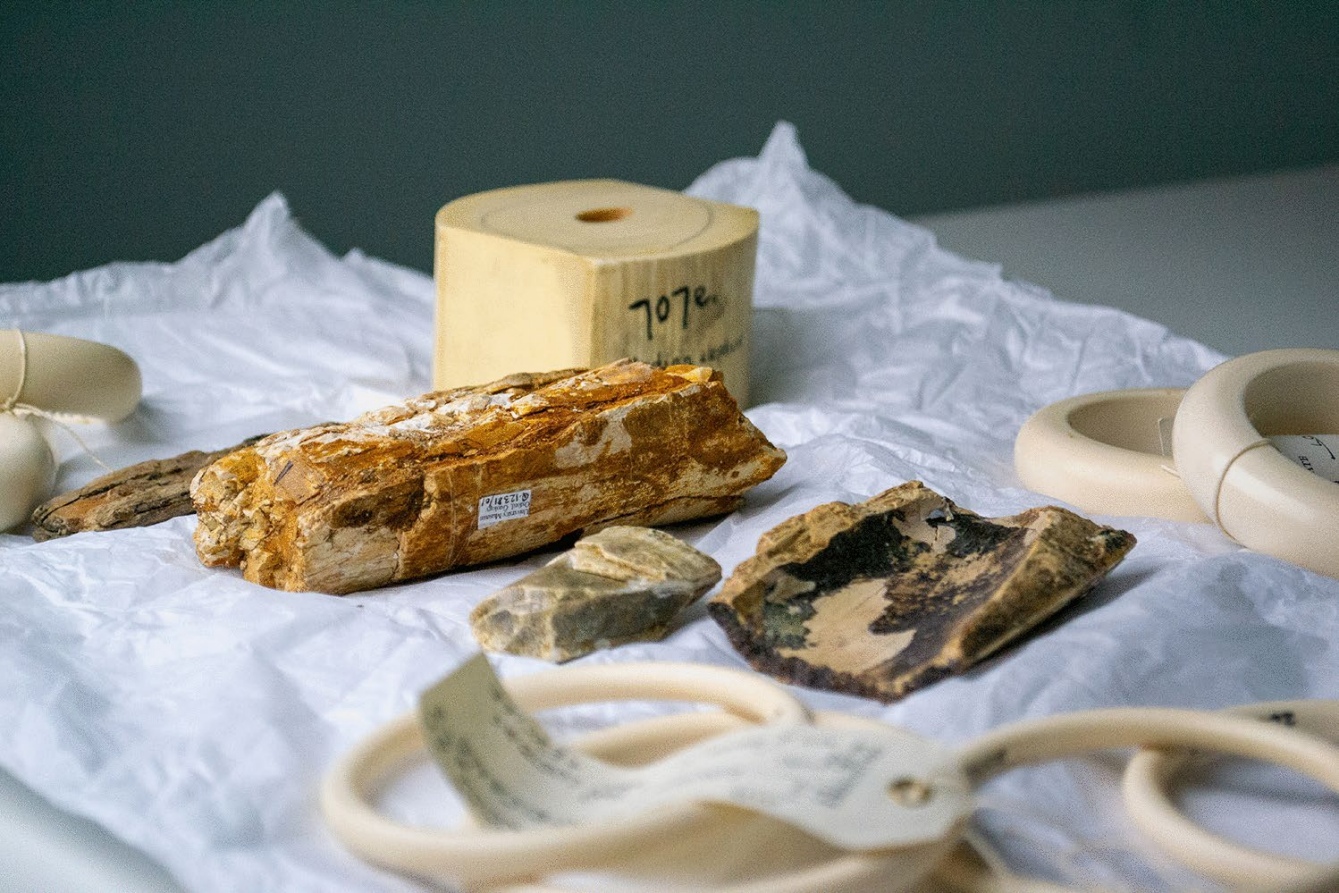
A small selection of ivory specimens from extinct and extant elephant species that were included in the analysis.

**Fig. 3:**
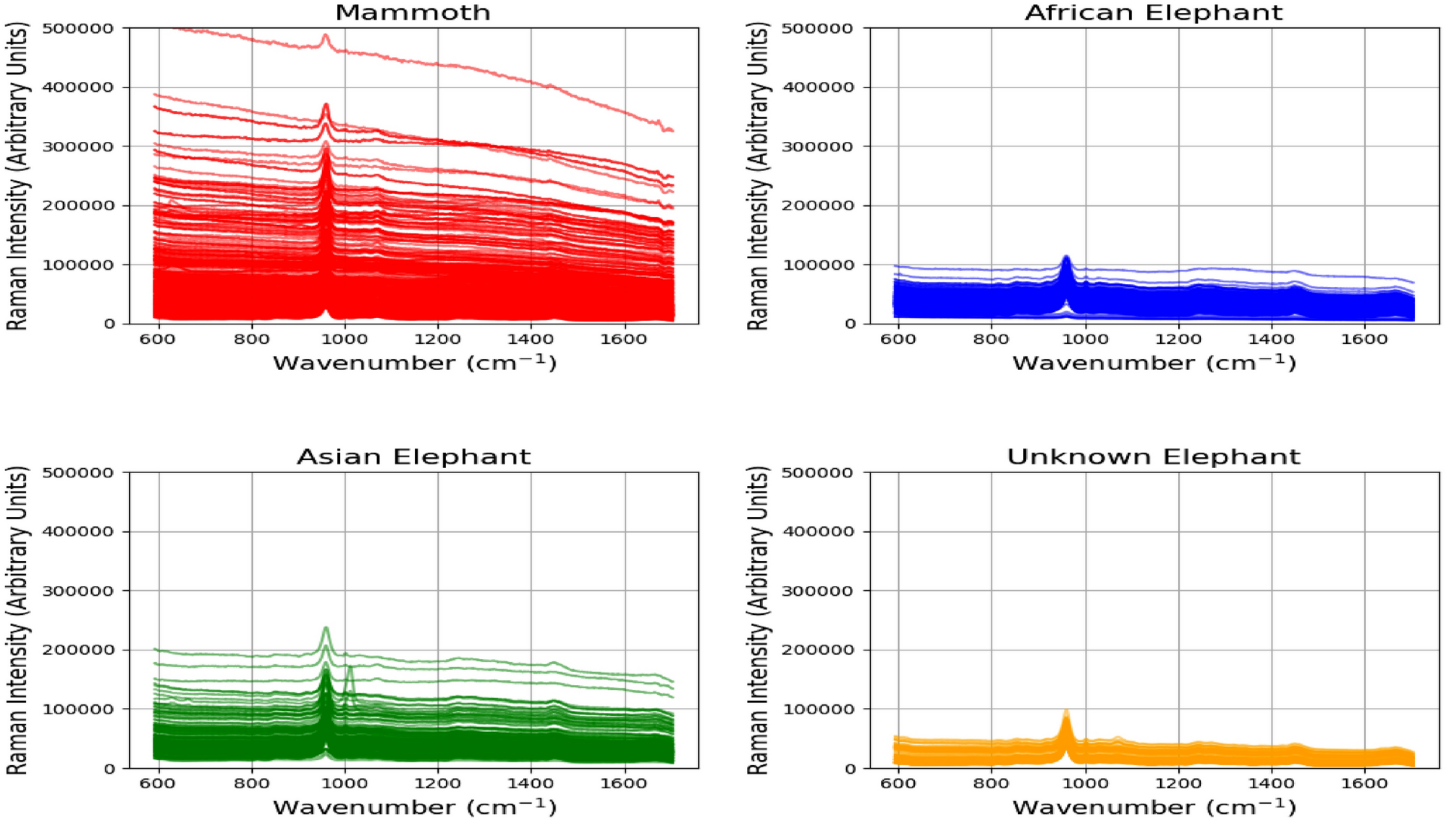
Sample spectra for each subclass in the Ivory Classification Dataset.

The ivory Raman spectra dataset consists of 822 Raman spectra (wavelength range 593–1704 $\hbox {cm}^{-1}$) taken from a total of 38 discrete ivory samples from species *Elephas maximus*, *Loxodonta spp*, *Mammuthus primigenius, Mammuthus trogontherii *and* Palaeoloxodon antiquus.* Figure 2 demonstrates a small number of ivory specimens from extinct and existing elephant species included in our study. Spectra were acquired from each ivory sample using an inVia Raman micro spectrometer (Renishaw Ltd, Gloucestershire, UK) equipped with an Olympus $50 \times$/0.5 long working distance objective lens and a 785 nm laser with a 200 mW at source and 1200 l/mm grating. The laser power at the sample was approximately 10 mW. Each spectrum was collected for 60 s ($6 \times 10$ s accumulations) in the spectral range $\tilde{6}00$–1700 $\text {cm}^{-1}$, which are represented in Fig. 3 with corresponding plots for each subclass separately. Spectra were collected at 10 different locations on each sample to provide replicates and to account for material heterogeneity. The samples used in this study were kindly loaned from the Natural History Museum (London, UK), Oxford Museum of Natural History (Oxford, UK) and Lancaster City Museums (Lancaster, UK). Table 1 describes the statistics of the Ivory Classification Dataset by the total number of spectra for mammoth and 3 subclasses of elephant samples (Asian Elephant, African Elephant, and Unknown Elephant).

**Table 1:** The statistics of the ivory classification dataset.

Sample	Number of spectra
Mammoth	370
Asian Elephant	149
African Elephant	217
Unknown Elephant	86

#### Machine learning Raman Open Dataset (MLROD) dataset

MLROD dataset^12^ contains about 500, 000 spectra across 16 classes with wavenumbers from 150 to 1100 $\hbox {cm}^{-1}$. Table 2 demonstrates the mineral dataset, containing various types of (inorganic) minerals from a number of locations throughout the world.

**Table 2:** The statistics of the machine learning Raman Open Dataset (MLROD)^12^.

Sample	Location	Number of spectra	Dataset
Gabbro slab—0% dust	Madagascar	8952	Test
Gabbro slab—50% dust	Madagascar	9740	Test
Granite slab—0% dust	Unknown	11,028	Test
Granite slab—50% dust	Unknown	10,000	Test
Hawaiian Basalt Dust	Big Island, HI, United States	–	–
Quartz	Spruce Pine, NC, United States	10,032	Training
Feldspar–Albite	Bancroft, ON, Canada	5875	Training
Feldspar–Anorthite	Miyaka-jima, Tokyo, Japan	5600	Training
Feldspar–Microcline	Fremont, CO, United States	5600	Training
Amphibole–Hornblende	Dresden, SN, Germany	5064	Training
Mica–Biotite	Bancroft, ON, Canada	7925	Training
Mica–Muscovite	Ottawa, ON, Canada	5900	Training
Olivine–Fosterite	San Carlos, AZ, United States	5900	Training
Pyroxene–Augite	Trento Province, Italy	6103	Training
Pyroxene–Enstatite	Mpwa, Tanzania	5600	Training
Carbonate–Calcite	Unknown	5032	Training
Sulfate–Gypsum	Unknown	5000	Training
50% Quartz/50% Albite mix	Powder mix from locations above	5000	Training
50% Forsterite/50% Augite mix	Powder mix from locations above	5040	Training
50% Fosterite/50% Albite mix	Powder mix from locations above	5220	Training

### Experimental setup

All experiments were conducted on a laptop equipped with 32 GB of RAM and an NVIDIA GeForce RTX 3080 GPU with 16 GB of memory. This hardware setup provided sufficient computational power to handle the datasets and execute machine learning models used in this work.

#### DL baseline

DL baseline built upon the architectures pretrained on MLROD dataset (as further described in Sect. 4). The hyperparameters of MLROD pretraining included batch size 20, a learning rate 0.01, dropout rate 0.55, with the optimisation process duration of 150 epochs.

#### PCA/LDA Baseline description

Initially, PCA was performed for dimensionality reduction purposes. For comparison, we varied the number of principal components between 1 and 100 to explore the trade-off between data simplification and loss of informative variance. Following the PCA, Linear Discriminant Analysis (LDA) was used to project the data onto a new axis, maximizing class separability between two classes of ivory, elephants and mammoths. The LDA implementation used default settings of discriminant_analysis.LinearDiscriminantAnalysis from sklearn Python library, which included singular value decomposition as the solver (solver=’svd’). Cross-validation for PCA/LDA was conducted using a six-fold split.

#### Data preprocessing

The preprocessing steps for the PCA/LDA baseline and for the proposed DL models included dimensionality reduction, normalisation, and wavenumber adjustments. These steps are described below.

#### Data preprocessing for PCA/LDA analysis

The input data standardisation has been carried out per-feature using the following equation: $z = \frac{x - \mu }{\sigma }$, where $x$ is the original feature value, $\mu$ is the mean of the feature, and $\sigma$ is the standard deviation.

Principal Components (PCs) (number of components from 1 to 100) were employed on the ivory dataset. This preprocessing step was crucial to reduce dimensionality and to focus on the most informative aspects of the spectra. The PCA model was implemented using sklearn’s function sklearn.decomposition.PCA, which included not whitening the components (whiten=False) and automatically selecting the algorithm for singular value decomposition (svd_solver=’auto’).

#### Data preprocessing for DL

The input sequence length was fixed to 950 dimensions according to the preprocessing steps in^21^. The wavenumber data in the training and testing sets were trimmed to a range of 600–1640 $\hbox {cm}^{-1}$.

### Experimental results

Results demonstrate the advantages of the proposed method against the state-of-the-art baseline. The recently published work on the use of Raman spectroscopy for ivory identification^6^ used PCA with subsequent use of LDA for classification of time series. Table 3 shows that the methodology described in this paper provides better accuracy over the previously used statistical analysis^6^, with up to $99.7\%$ against $96.2\%$ for PCA/LDA. When using the IDEAL method^34^, three different clustering techniques were used to identify prototypes; ELM, mean shift and *k*-means. This corresponds to both, full finetuning of the model on the target data and merely linear finetuning of the pretrained model (as discussed in Sect. 4). The accuracy and confidence intervals for xDNN and IDEAL methods are reported using ten-fold cross-validation.

For the methods that do **not** require finetuning, only the training data set (MLROD) was used to identify prototypes through clustering^28,34^. While these results are lower ($92\%$) than the ones obtained after supervised finetuning, it still have the potential aid in the identification of unknown ivory samples, and demonstrates that labeling the ivory data is not necessarily required for training/finetuning.

**Fig. 4:**
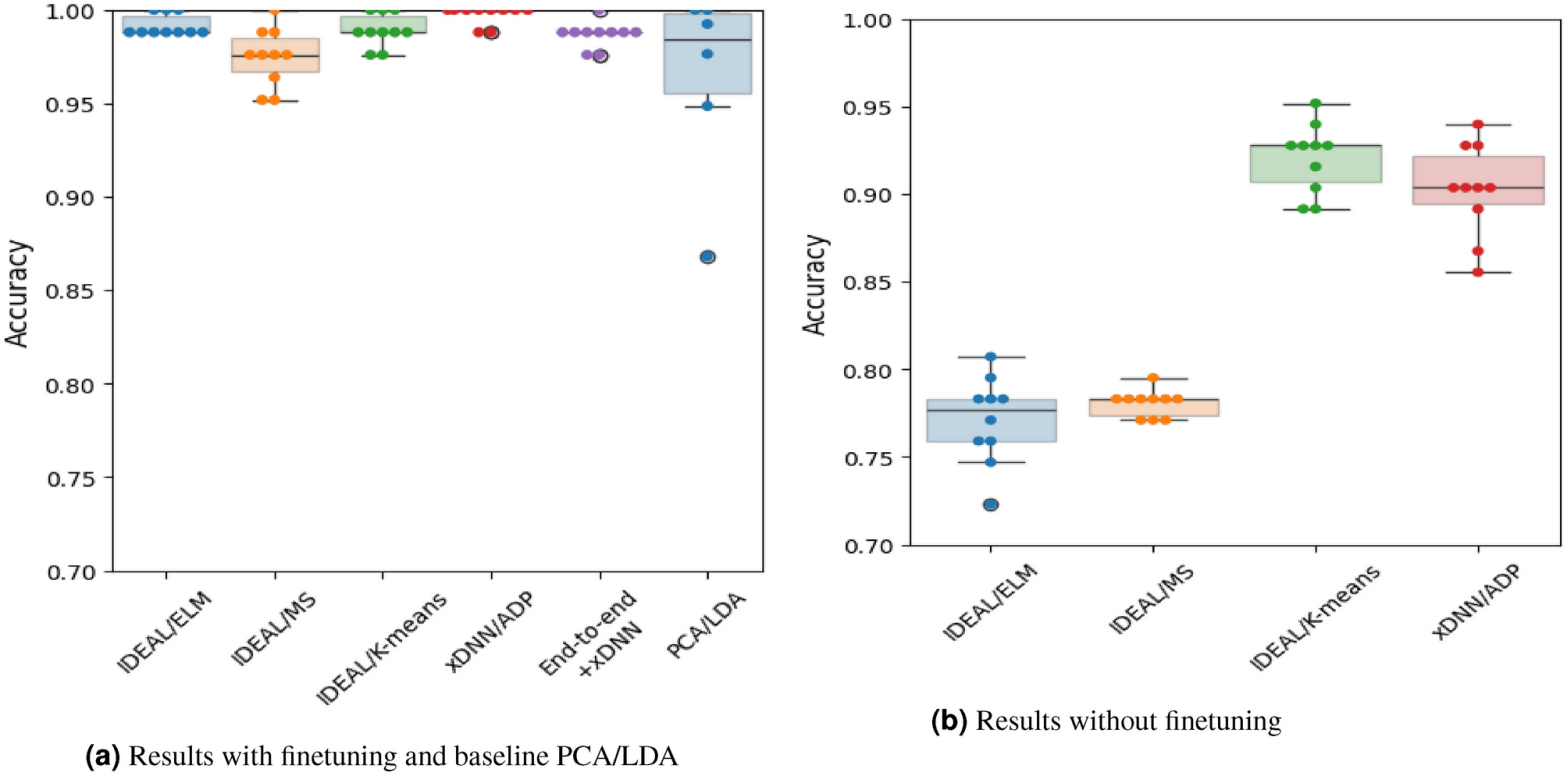
Comparison of results for xDNN and IDEAL, and the LDA baseline.

Figure 4 demonstrates that for a number of settings, notably IDEAL/*k*-means and xDNN/ADP, one can obtain consistently good performance. It is also observed that the models with no pre-defined number of clusters, such as ADP, can perform competitively with the methods requiring prior knowledge of the number of clusters, such as *k*-means.

**Table 3:**
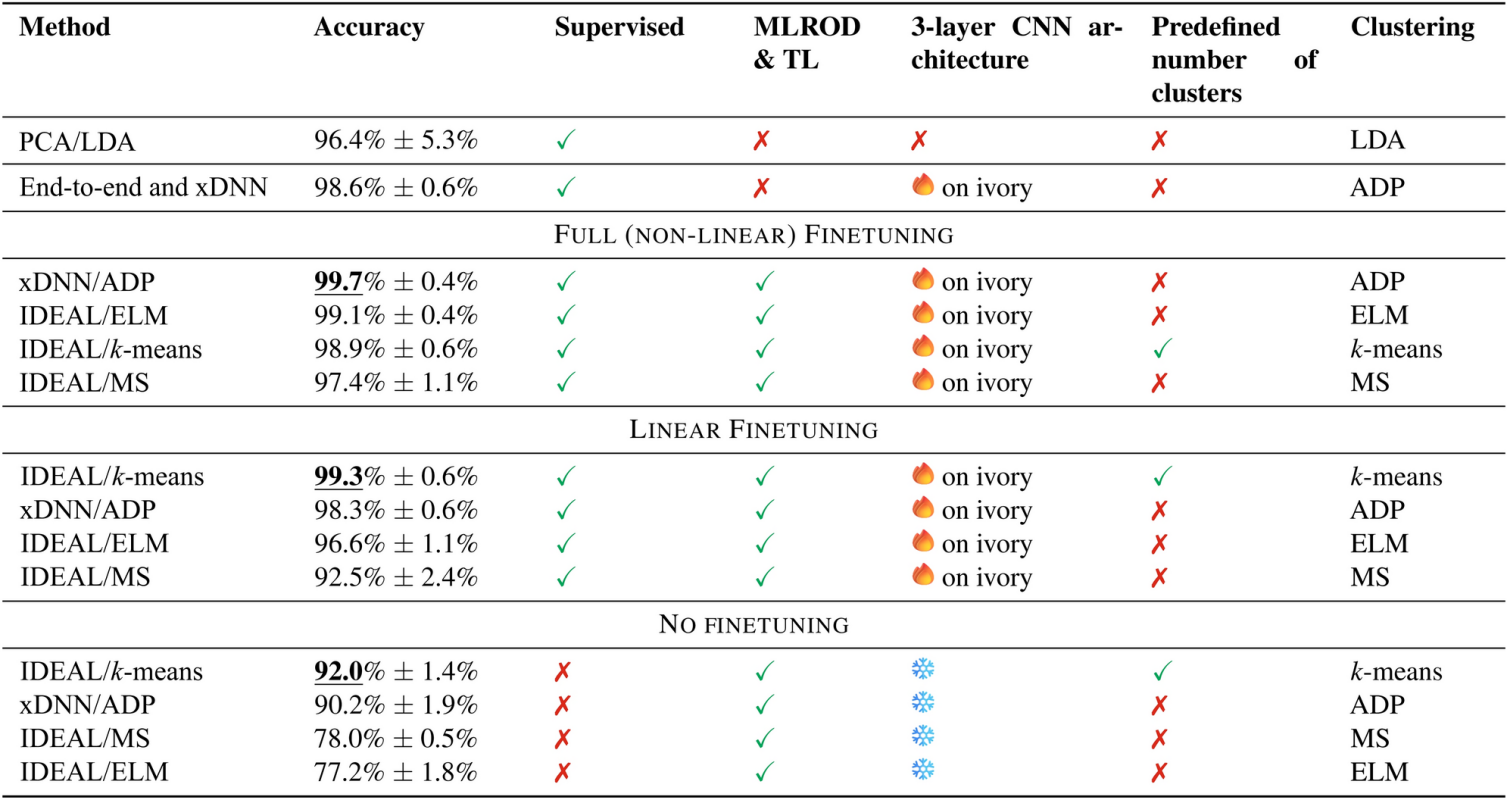
Comparative performance between PCA/LDA and interpretable deep-learning methods; MS denotes mean shift clustering.

Significant values are in bold-underline.

## Discussion

### From stones to bones: transferring from inorganic to organic data

TL has been instrumental in leveraging high performance of the ML models in domains such as vision and natural language processing^32,35^. It has been shown that TL methods have been applied to a number of use cases in studies such as bioinformatics^36^ and spectral analysis^37^ amongst others. In this work, we leverage existing Raman spectroscopy data of geological samples, available in significantly large, open source data sets (such as MLROD^12^) to classify biological ivory data, both with and without any further training.

For completeness of the experiment, full (end-to-end) training was also applied to the ivory training data set. This used the same settings (xDNN on top of the 3-layer CNN), but without priming it with the MLROD data set and without using TL. The results, shown in Table 2, were $98.6\%$. This value was lower than the results of $99.7\%$ when TL was used. It additionally required a labeled training ivory data set to be provided, unlike the experiment which achieved $92.0\%$ accuracy.

These results show that despite the significant differences between the chemical compounds of the geological samples and the ivory, TL helps achieve higher accuracy ($99.7\%$ versus $98.6\%$) and, remarkably, even without any supervised training on labeled ivory data at all it offers accuracies of up to $92\%$. This paper presents the first, to the best of our knowledge, evidence which that TL from inorganic materials can be very efficient to apply to biological ivory data identification.

### Interpretation and insight into the decision making process

In the proposed method, the prediction is performed using prototypical spectra derived from the training data. This helps not only achieve high performance but also provide insight into the decision making within the proposed model.

**Fig. 5:**
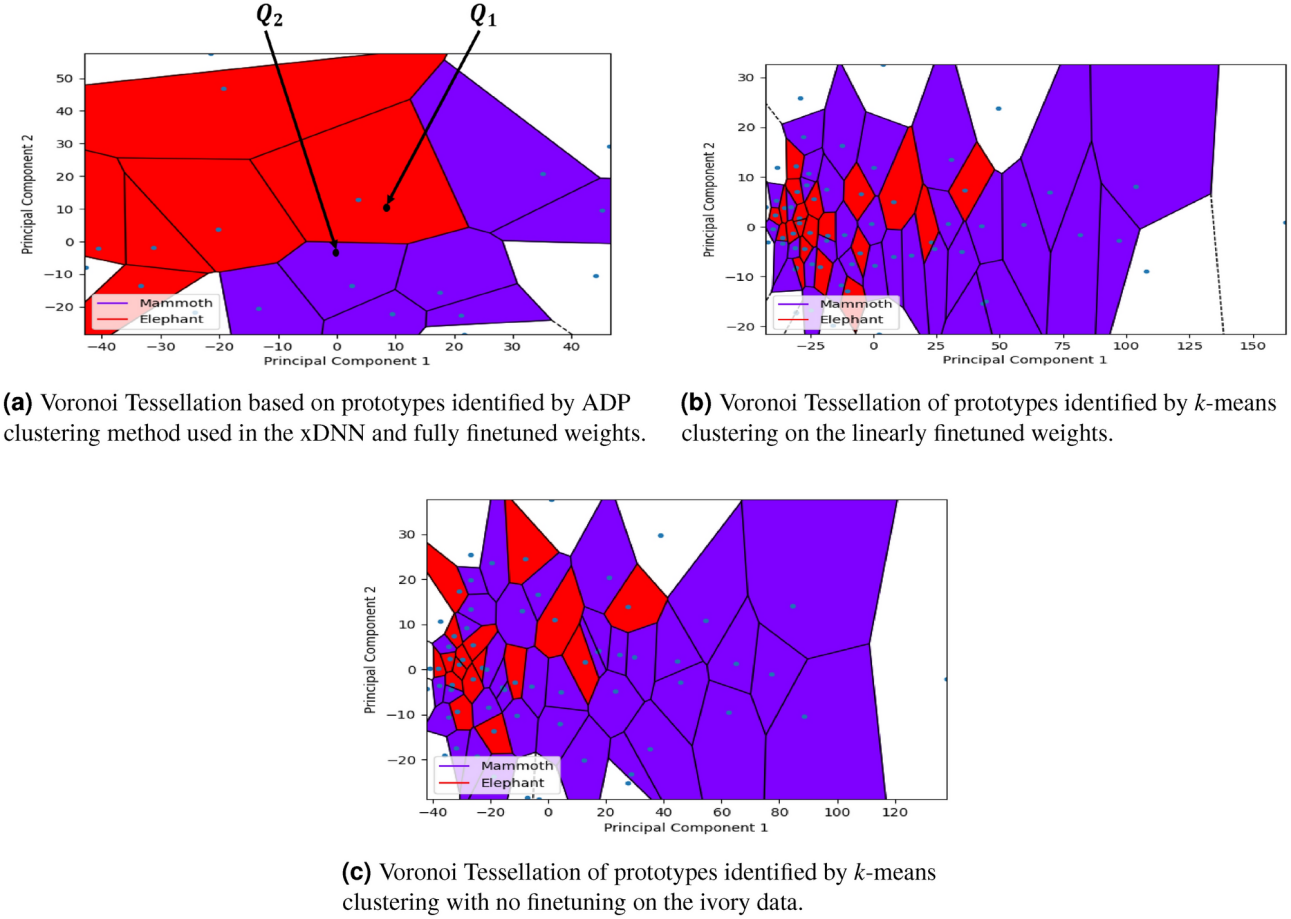
Voronoi Tessellations of the identified prototypes in three different scenarios (full finetuning, linear finetuning and no finetuning) on the case of two classes.

Figure 5 visualises the identified prototypes in these scenarios which form a so-called Voronoi tessellations using the same data, but combining all different types of elephants into an aggregated class “Elephant” to simplify the color scheme and the visual effect. Figure 5a shows that the finetuned version of the proposed method results in a significantly clearer separation between the two classes. The 2D PCA plot also demonstrates how the labels of queries ($Q_1$, $Q_2$) are determined based on the distance to nearest prototype. For example, we consider $Q_1$ to be assigned a label of “Elephant” and $Q_2$ - the label “Mammoth”, respectively.

**Fig. 6:**
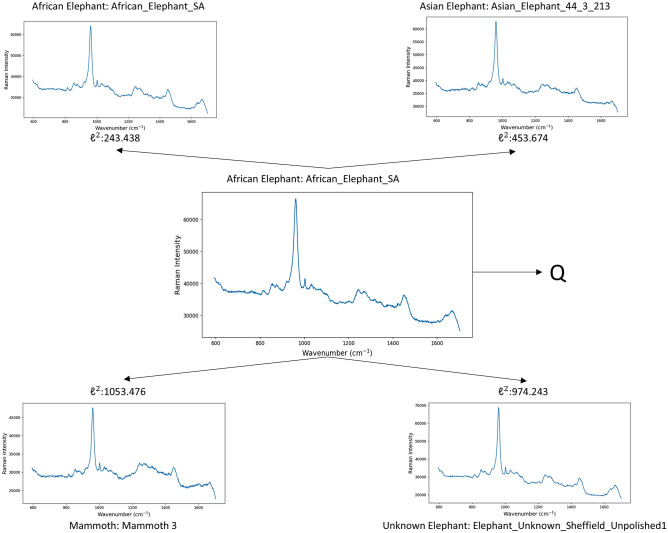
Interpreting the prediction for the query sample *Q* named “African_Elephant_SA” by identifying the nearest prototypes for each class or subclass (Elephant class has 3 subclasses - African, Asian, and Unknown). The nearest prototype to that sample belonged to an African Elephant (“African_Elephant_SA”) sample, so it is correctly classified as an Elephant.

**Fig. 7:**
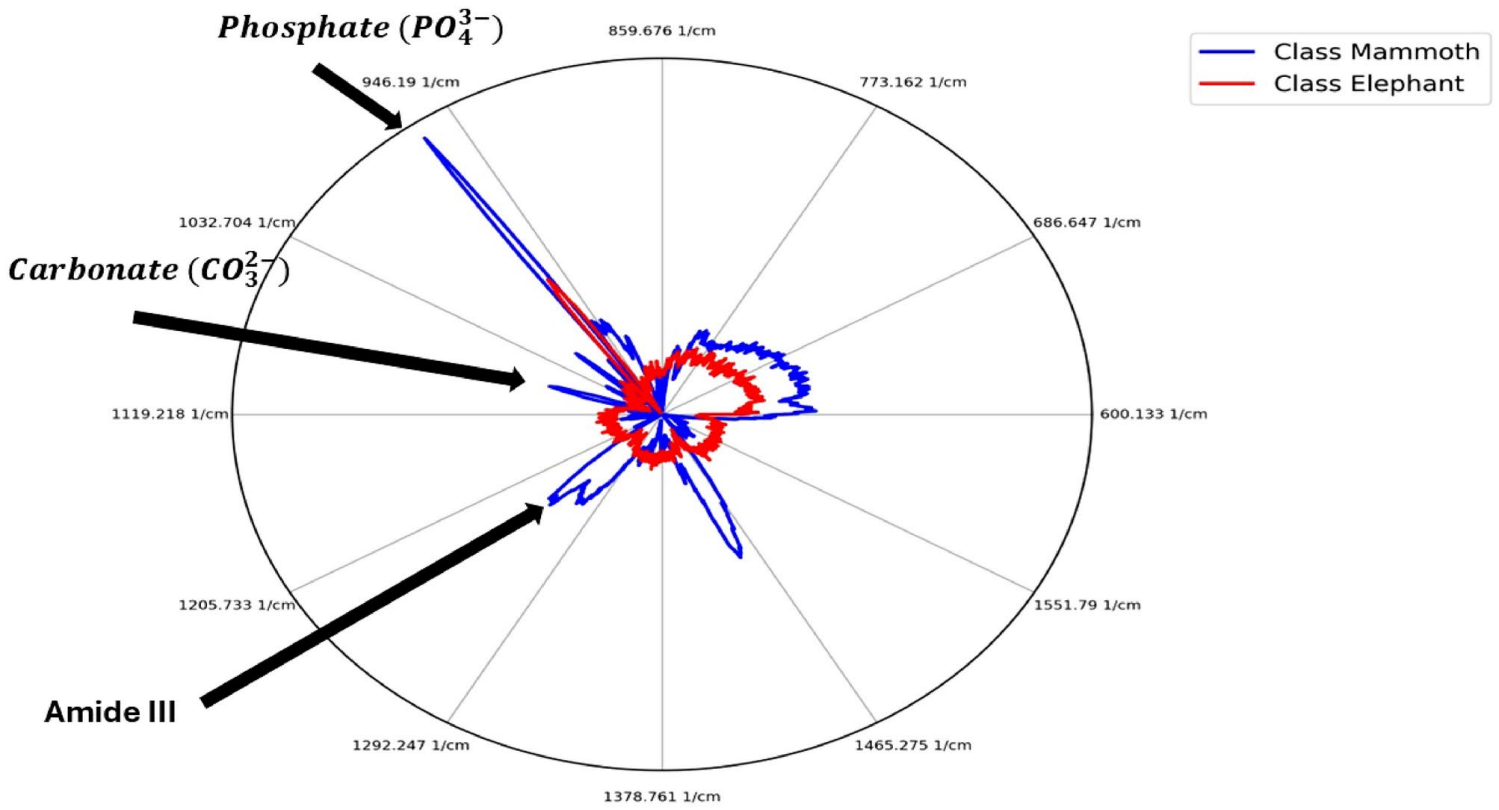
Interpreting the prediction for the query sample that belongs to the Elephant class by identifying the nearest prototypes for each class. The radar plot shows the distances between a query sample and the nearest prototypes of two classes, Mammoth (blue) and Elephant (red), across all wavebands. The query sample is classified as Elephant due to the shorter distance over all wavenumbers.

Figures 6 and 7 demonstrate how the proposed method is able to both, interpret the results and guide the decision making process. Figure 6 provides the prototype-based interpretability for a query sample with its nearest prototype for each class or subclass. While the details of the proposed approach are described in “Methods”, Fig. 6 shows the closest prototype in terms of their $\ell ^2$ distance which is pivotal to determine the winning class label. In the figure, the Raman spectra which represent the raw data is presented for five cases: (1) the query sample for which the class label is unknown and we like to determine it; (2) the nearest prototype from each class or subclass. Obviously, one of these is the winning class which, in this case, is African Elephant sub-class. Such form of visualisation is easy to interpret by a human user and also allows to analyse further cases of errors, details such as waenumber-absorption pairs, etc.

Figure 7 is a radar plot which allows to visualise the decision made in a different form with more clarity per feature (wavenumber which, in turn, corresponds to a particular biochemical compound). In Fig. 7, for example, the plot visualises three data items (spectra): (1) the query (the spectrum we would like to classify); (2) the closest one from each class (in this case we only consider two classes with no sub-classes for simplicity—mammoth and elephant aggregating all types of elephants together). Within the radar plot, the origin of the coordinate system (point (0, 0)) represents the query. The closer to the centre, the line is more similar to a prototype and vice versa. For this particular case, a label “Elephant” is assigned because the red curve is closer to the origin overall, but the radar plot allows to analyse this overall conclusion on a per-feature basis. The comparison of the Raman spectra of mammoth and elephant ivory, shown in Fig. 7, revealed several biochemical differences, including the phosphate peak (9610 $cm^1$), carbonate peak (1070 $cm^1$), amide III peaks (1250 and 1270 $cm^1$), and CH2 (1454 $cm^1$). These findings are consistent with previous work using PCA and associated loading plots to identify similar biochemical variations^6^. The mammoth ivory analyzed in this study consisted of specimens that were both well-preserved and taken from the permafrost and more degraded samples excavated from quarries in the UK. For the degraded specimens, degradation of organic components would be expected, particularly from the protein regions of the spectra. Ivory preserved in permafrost typically has a higher collagen-to-mineral ratio compared to fresh ivory^38^. Other factors, such as dietary differences, climate conditions, and post-mortem burial conditions, may also affect the biochemistry of the tusk. Studies on buried bone samples show evidence of post-mortem alterations, where trace elements are exchanged between the bone and its surrounding environment^39^. Within biological apatite, there are a number of elemental substitutions that can occur, including anionic (F−, Cl−, SiO44−, and CO32−) and cationic substitutions (Na+, Mg2+, Fe2+, K+, Sr2+, Zn2+, Ba2+ and Al3+). The proposed approach not only offers a high accuracy, but can also be used as a tool and mechanism for a more detailed analysis of the decision (including of wrong classifications).

### Implications for the ivory trade

Innovative methods in ivory identification could be key in tackling the illegal ivory trade and to prevent the decline of wild elephant numbers. This work demonstrates two ways in which this research could help this purpose: (1) the highly accurate ($99.7\%$) classification of ivory species could enable the timely detection of illegal ivory specimens, giving a more accurate result compared to previously used PCA/LDA analysis for discrimination of ivory Raman spectra, as well as the end-to-end training on labeled ivory data (2) confident ($92\%$) prediction of source of unknown ivory which can help discover the source of data in face of lack of knowledge about the sample origin, even without finetuning, which means labeling, experimenting and significantly higher software and hardware requirements including computing/ML expertise.

Current gold standard techniques in ivory identification include visual morphological inspection of a sample by an expert, DNA analysis or radiocarbon dating, all of which are expensive and time-consuming, and the latter two techniques are destructive. The use of Raman spectroscopy, coupled with ML and TL, in particular, offers a methodology in ivory detection that could be used as an initial assessment of ivory before more expensive and destructive methods are used.

## Methods

### Deep neural network trained on MLROD data

**Fig. 8:**
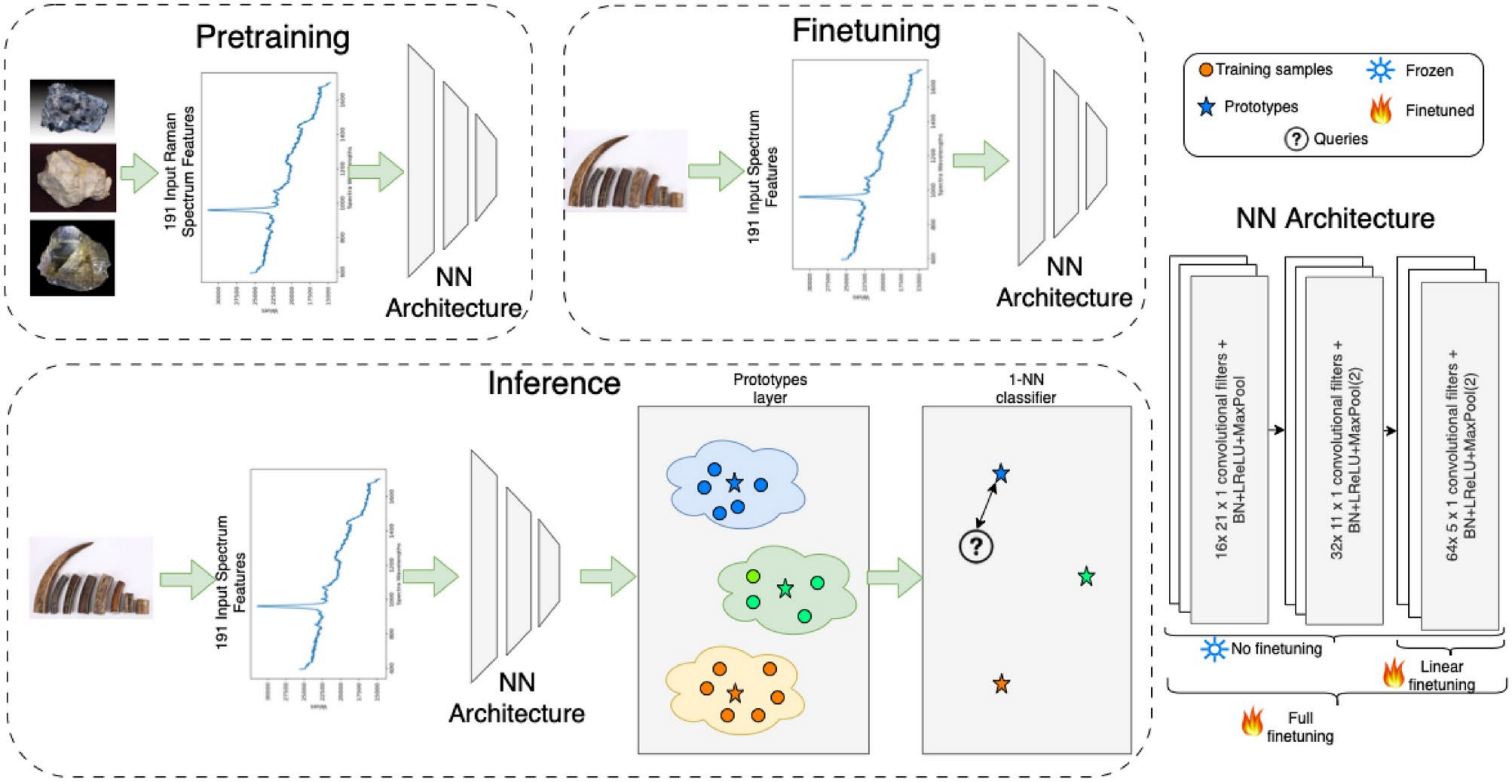
The proposed framework for ivory analysis.

Following^21^, a convolutional neural network (CNN) architecture was used (see Fig. 8) with three one-dimensional convolutional layers, each followed by a batch normalisation layer and a one-dimensional max pooling layer. The predictions were performed through a prototype layer, followed by a nearest neighbour classifier. The prototypes were identified using different clustering methods (ADP^28^, *k*-means^40^, Mean-shift^41^, ELM^42^) and selecting the closest real data sample spectra to the cluster centroid in a medoids-like style^43^.

The feature extraction process involved utilising the three convolutional layers of the CNN architecture, the same as used in^21^. Once each spectroscopy sample has been processed through these layers, the output was flattened to create 2048-dimensional (2048D) feature vectors. These vectors represented a comprehensive encoding of the spectral data, capturing essential patterns for detailed further analysis by the proposed framework.

For all experiments, except the end-to-end + xDNN (second row of the Table 3), the CNN was pretrained on the MLROD dataset^12^ using the procedure outlined in^21^, using the settings defined in Sect. 2. The model has been evaluated in three different scenarios, described below: full finetuning, linear finetuning and no finetuning.

#### Full finetuning on ivory data

In this scenario, the entire network has been finetuned on the training set of the ivory spectral data. The hyperparameters set for this phase were a learning rate of 0.001, a batch size of 20, a dropout rate of 0.55, and the training was extended over 250 epochs. These parameters were carefully chosen to ensure a thorough learning process without overfitting, maintaining the balance between model complexity and performance accuracy.

#### Linear finetuning using ivory data

Linear finetuning was applied only to the last convolutional layer (the last layer of the feature extractor), utilising the same hyperparameters as the full finetuning method. This strategy was employed to fine-tune based on the more specific features of the ivory dataset while preserving the integrity of the generalised model learned during pretraining on MLROD dataset with frozen parameters.

### Prototype-based deep neural network classifiers

#### xDNN

The xDNN model^28^ is an interpretable-through-prototypes method which provides interpretations in terms of proximity in the latent space, Voronoi tessellations as well as rule-based systems.

The model performs the following steps:

1. Inference using a frozen model, trained on generic or target data: in our case, it is the CNN pretrained on MLROD dataset and/or finetuned on target data as per Sect. 4.
2. Density and typicality layers, representing, respectively, the prototype distributions and its score for prototype selection.
3. Prototypes layer, which selects prototypes by the local peaks of typicality using ADP clustering method^44^.
4. Decision making process through interpretable rule-based system.

#### IDEAL

The IDEAL model^34^ has a similar scope to xDNN, however it does not calculate density or typicality layers. Instead, it performs prototype identification through clustering of data and selecting closest training sample. It performs classification through nearest-neighbour classification.

It uses the following optimisation problem^34^:

1 $$\begin{aligned} \arg \min _{\varvec{\theta }_{d}} \sum _{({\textbf{x}}, y) \in ({\mathbb {X}}, {\mathbb {Y}})} l (h (d ({\textbf{x}}, {\textbf{p}} ; \varvec{\theta }_d)|_{p \in {\mathbb {P}}}), y). \end{aligned}$$

where ${\mathbb {X}}, {\mathbb {Y}}$ are respectively inputs and labels, and ${\mathbb {P}}$ is a set of prototypes derived from data ${\mathbb {X}}$ (e.g., by selecting a set of representative examples or by clustering).

The distance $d(\cdot , \cdot )$ is chosen to be the negative Euclidean distance:

2 $$\begin{aligned} d ({\textbf{x}}, {\textbf{p}} ; \theta _d) = -\ell ^2 (\phi ({\textbf{x}} ; {\varvec{\theta }}_d), \phi ({\textbf{p}} ; {\varvec{\theta }}_d)), \end{aligned}$$

where $\phi$ is the feature extractor output as per Sect. 4 and ${\varvec{\theta }}_d$ are its parameters. For the scenario without finetuning, ${\varvec{\theta }}_d$ is frozen: $\phi (\cdot ) = \phi (\cdot ; {\varvec{\theta }}_d), {\varvec{\theta }}_d = \textrm{const}$. The similarities bounded between (0, 1] could be obtained by, for example, taking the exponential of the similarity function or normalising it. The function *h* is a winner-takes-all operator:

3 $$\begin{aligned} h (\cdot ) = \textrm{CLASS} (\arg \min _{p\in {\mathbb {P}}} d(\cdot , {\textbf{p}} ; \theta _d)) \end{aligned}$$

### Code availability

The code supporting this work is included in the Supplementary Information files submitted alongside this manuscript.

## Conclusion

This paper clearly demonstrates the ability of ML and, in particular, DL methods to provide highly accurate techniques (up to 99.7%) in the differentiation between Raman spectra of ivory from extant and extinct species of elephant. This has the potential to aid in stopping illicit trade, through the development of quick and inexpensive testing that is not reliant on experts in the field. The proposed method takes advantage of widely available geological Raman spectroscopy data, demonstrating how it can be leveraged through TL to get highly accurate identification of biological ivory data. The accuracy is higher compared to an end-to-end training using deep learning with no TL, as well as compared to the previously used PCA-LDA method in classifying Raman spectra from ivory samples. TL alleviates the need for labeled ivory data by offering quite impressive accuracy of $92\%$ using a DL model trained only on Raman spectroscopy data of inorganic minerals combined with TL and no further training/finetuning.

This demonstrates the ability to classify unknown sources of ivory with performance above $92\%$ without the need for a large, pre-existing ivory specific dataset for training. Furthermore, this paper demonstrates the insight into the decision making process using prototype-based models and visualisation. The emphasis on the region of spectra was responsible for variation in chemical structure of ivory from difference elephant species, enabled the analysis of decision making into biochemical components that correspond to these wavenumbers.

To the best of our knowledge, this is the first publication to report: (1) the application of deep learning (and machine learning) for ivory identification; (2) high accuracy levels that would enable practical use in combating illegal ivory trade; (3) transfer learning from inorganic materials to biological data; and iv) insights into the decision-making process of deep learning models through prototype-based approaches.

Going forwards, this methodology, coupled with Raman spectroscopic analysis, has the potential to be used to classify ivory from other animal species. It could have potential to be used on other mineralised tissues, for example in identifying disease states within human bones and cartilage.

## Supplementary Information

Supplementary Information 1.

Supplementary Information 2.

## Data Availability

The Ivory Classification Dataset used in this work is included in the Supplementary Information files submitted with this article.
